# Landscape of Digital Technologies Used in the National Health Service in England: Content Analysis

**DOI:** 10.2196/51859

**Published:** 2024-04-19

**Authors:** Jake Alan Allcock, Mengdie Zhuang, Shuyang Li, Xin Zhao

**Affiliations:** 1 Department of Sociological Studies University of Sheffield Sheffield United Kingdom; 2 Information School University of Sheffield Sheffield United Kingdom; 3 Business School University of Birmingham Birmingham United Kingdom

**Keywords:** digital health, healthcare service, regional difference, National Health Service, NHS, digital technology, health equity

## Abstract

**Background:**

In England, digital technologies are exploited to transform the way health and social care is provided and encompass a wide range of hardware devices and software that are used in all aspects of health care. However, little is known about the extent to which health care providers differ in digital health technology capabilities and how this relates to geographical and regional differences in health care capacities and resources.

**Objective:**

This paper aims to identify the set of digital technologies that have been deployed by the National Health Services clinical commissioning groups (NHS CCGs) in England. In doing this, we respond to calls to shed light on the internal dynamics and variation in the form of digital capability in England in terms of health service regional differences and health diversity, equity, and inclusion.

**Methods:**

We collected 135 annual reports that belong to 106 NHS CCGs in England, comprising more than 18,000 pages in total, released from 2020 to 2021. Using this data set, we identified 2163 pages related to digital technologies and labeled them using content analysis. We follow the construct taxonomy used by digital options theory, a theory from the management information systems field analyzing organizational resource investment choices, in classifying observed technologies according to digital *themes*—inherent design patterns that we identified and explained. We then used a hierarchical clustering method to extract groups of NHS CCGs that implement similar technology themes.

**Results:**

We found 31 technologies from the reports and grouped them into 9 digital themes. The 9 themes were further assigned to 1 of the 3 constructs of digital options theory, *the identification of patients’ requirements* (we identified information portals [76/106], digital health engagement [67/106], and digital inclusion support [45/106]), the *development of new work patterns* (we identified telehealth [87/106], telemedicine [35/106], and care home technologies [40/106]), *the realization of improvements in efficiency and public accessibility* (we identified online booking [26/106], online triage [104/106], and digital mental health services [74/106])*.* The 3 clusters of CCGs are identified based on the 8 themes (Hopkins=0.9914, silhouette=0.186), namely (1) digitally disengaged, (2) digitally engaged, and (3) digital torchbearer.

**Conclusions:**

Our findings show prominent digital themes within each construct group, namely information portals, telehealth, and online triage, covering people’s fundamental health information needs. Almost half of CCGs fell into the digitally disengaged group, and all London CCGs (5/106) belonged to this group. We propose that practitioners should offer specialized assistance to regions with limited digital engagement, emphasizing digital health literacy, inclusion support, and ongoing evaluation, rather than concentrating solely on technical advancements.

## Introduction

The health care services in England are in a transformational phase due to the increasing pressure to propose and review digital strategies, use continually emerging new digital technologies, and place varied emphasis on digitization according to regional needs [1,2]. Digital technologies are exploited to transform the way health and social care is provided and encompass a wide range of hardware devices and software that are used in all aspects of health care. These technologies are often grouped and examined by their use cases [3,4], such as medical consultations and treatment, patient management, and information campaigns for access to care. Some researchers emphasize the change these technologies may bring (also referred to as “innovation”) and therefore propose a different type of taxonomy. Zweifel [5] categorized technologies serving the purpose of health care innovation into 3 types, including technologies for product innovation, process innovation, and organizational innovation.

Despite these developments, however, health care practitioners and managers often find it difficult to choose and leverage different forms of digital technologies and innovation in their institutions to overcome challenges or inefficiencies [6]. Furthermore, little is known about the extent to which health care providers differ in technology capabilities regarding improving public access to health resources, and how this relates to geographical and regional differences in health care capacities and resources. In the United Kingdom, the availability and usage of medical treatments (measured by, eg, travel distance to attend treatment, waiting time to receive a treatment, and funding for a primary-care practice) vary across regional health services in England [7-10]. We suspect this variation may also apply to the availability of digital technologies as a result of a local strategy priority, prior digitalization level in the area, and the demographics of the local populations.

Drawing from this emerging stream of research, this study identified the digital technologies adopted in 106 National Health Service clinical commissioning groups (NHS CCGs) in England, using 135 annual reports for the years 2020-2021. The CCGs were clinically led statutory NHS bodies responsible for the planning and commissioning of health care services for their local area, which were dissolved in 2022 to be replaced by the new integrated care boards (ICBs). The CCG annual reports were selected for 2 main reasons. First, the CCGs’ structure (106 CCGs in England as of April 2021) provided a finer spatial granularity than the ICBs’ structure (42 ICBs in England as of July 2022). Therefore, they offered crucial insights and statistics for all involved NHS services across different regions, which could offer valuable information regarding differences in digital capabilities and diversity, equity, and inclusion issues across various fine-grained geographical sites. Second, the years 2020-2021 marked an important step in health care digital transformation as the CCGs afterward focused on structural change and were replaced by ICBs nationally. In addition, in response to the COVID-19 pandemic, CCGs were incentivized to make services accessible through digital platforms, aiming to ensure the continual provision of care while mitigating transmission risks. Therefore, the reports in 2020-2021 highlighted important aspects of digital technologies and themes adopted by the CCGs before the structural shift and in response to the COVID-19 pandemic, which were fed into the newly formed ICBs.

We aim to answer the following research questions: What are the digital technologies adopted by CCGs to improve their services as well as widen public access and engagement? How do they vary across regions?

We use the construct taxonomy used by digital options theory [11] to understand the different types of technologies that are used in health care institutions, and how these options support health care providers’ ability to translate their resources into performance. In the management information systems literature, digital options represent an organization’s investment in and adoption of information technologies [12]. Such adoption, as Sambamurthy and colleagues argued [11], together with changes to the organization’s technological environment, impacts an organization’s information technology capabilities. Therefore, digital options are primarily used to examine the evaluation of information technologies [12,13] and to assess how the technologies and digital capabilities in an organization can be transformed into performance improvement [11,14].

Following this set of ideas, in the context of health care, digital options represent *opportunities* to use new technical tools and features that will increase the service quality, efficiency, and public accessibility. Previous work involving digital options theory and health care focuses on performance improvement, for example, how cost-effective information technology solutions can enhance the financial performance of resource-constrained hospitals [15]. Little effort has been invested to map out the existing set of digital technologies in health care using the taxonomy of digital options.

Drawing on the concepts of digital options theory [16,17], the technologies serving the purpose of health care efficiency can be grouped using three constructs: (1) *identifying* patients’ requirements that involve recognizing new technical features for the digital health service (such as online community engagement and digital champion events); (2) *developing* new work patterns that improve internal coordination and working process (such as artificial intelligence [AI] diagnostics and digital prescribing); and (3) *realizing* improvements in efficiency and public accessibility (such as virtual consultation and direct online booking). We use these constructs as the theoretical lens to categorize the digital tools used in various CCGs in NHS England.

This paper aims to identify the set of digital technologies that have been deployed by the NHS CCGs in England. This study has several contributions. First, it contributes to the health care literature by providing insights into how regional differences in technology, under an NHS system in England, can vary in their capabilities and efforts in implementing digital technologies. We empirically draw the landscape of digital transformation in health care by unpacking the role of NHS CCGs in adopting technologies and by presenting the regional differences in England. In doing this, we respond to calls to shed light on the internal dynamics and variation in the form of digital capability and patient engagement in England in terms of health service regional differences and health diversity, equity, and inclusion [18,19]. Second, we extend digital options theory to understand its application of constructs within a health care setting. We applied and examined the 3 key approaches, proposed by Rolland et al [12], for technological options to engage patients as technology users and to improve health care service quality. The study demonstrates the complex choices faced by health care providers: while they need to address large numbers of patient queries and appointments through actionable digital options, they are constrained by regional resources and digital capabilities. Similarly, while different types of digital options could offer health care providers with new opportunities to understand patients’ needs and coordinate workflows, the actual adoption of options varies significantly across different regions.

## Methods

### Overview

In this study, we followed a text-mining approach and content analysis method to analyze the research data. Specifically, we used a text mining approach to extract key paragraphs and texts from the 135 NHS CCG annual reports released for 2020-2021 identifying digital technologies. We further followed content analysis methods to manually code and categorize the extracted paragraphs and texts through the lens of the digital options theory [16,17]. We then clustered the CCGs based on the corresponding technologies to reveal the similarities between CCGs and regional differences in digital technologies’ availability.

### Data Selection

Secondary data sources, namely annual reports of each NHS CCG, were used in this paper. We decided to use annual reports as they cover each CCG’s performance and accountability in the period, ranging from performance analysis, progress on key initiatives, and public and patient involvement to actual spending, and are the most recent reports before the start of the ICB-forming stage. The reports were downloaded from individual CCG websites directly. In total, there were 106 CCGs across England as of April 1, 2021, which was reduced from 135 CCGs in 2020 with the merger of 38 CCGs into 9. This paper investigates the 135 annual reports, 18,667 pages in total, released from 2020 to 2021. We further group the results according to the 106-CCG structure as it reflected the most recent structure of the CCG systems for 2021-2022.

### Data Preprocessing

Given that each report contains 44-222 pages and is labor-consuming to go through manually, highlighting the relevant texts visually can support the researchers in locating useful information for content analysis more quickly. The reports were preprocessed using the semantic matching method [20], a text mining technique, to identify and highlight texts that are relevant to author-selected keywords. The processing was implemented in Python (Python Software Foundation).

First, reports from 10 CCGs were manually screened by the first 3 researchers independently, to gather the initial set of keywords. The selected 10 CCGs covered the main geographic regions in England. The whole research team had regular meetings during and after screening to discuss the expansion of or trimming the keyword set. Frequent and relevant keywords were excluded from the set if they might cover a much larger field, such as “digital tool.” To validate the set, the second author applied semantic matching methods using the proposed keyword set on the 10 reports and manually screened the reports again to make sure the relevant contents were highlighted appropriately. The final set contained the following words: “digital,” “technology,” “AI,” “Machine Learning,” “e-,” and “online,” together with their derivations. Using this method, 2163 pages containing selected keywords were highlighted in the 135 reports.

### Data Analysis

#### Content Analysis

After the preprocessing, we analyzed the extracted 2163 pages through content analysis. Content analysis [21] was conducted by the first author. According to digital options theory, there are three types of options when engaging with technologies, including: (1) *identifying patients’ requirements that involve recognizing new technical features for the digital health service* (eg, digital survey to gather patient feedback on services), (2) *developing new work patterns that improve internal coordination and working process* (eg, AI diagnostic tools), and (3) *realizing improvements in efficiency and public accessibility* (eg, appointment booking system). We first conducted the initial coding by going through the research data and grouping them into categories according to the purpose of the adopted technologies. Second, we went through the categories in detail and mapped them to the 3 options as themes. Regarding reliability, the research team had weekly meetings among all the authors throughout the analysis stage to constantly review the emerging codes and categories and to ensure agreement among researchers was reached. The codes assigned for each CCG can be found in Multimedia Appendix 1.

In total, 31 types of technologies were extracted, covering a wide range of tools and purposes, including online booking systems, virtual consultation, health care promotion campaigns, patient access to health care records, remote monitoring of patient status, AI diagnostics, mental health online service, and digital champions podcasts (see full list in Table 1). Further, 9 themes were then identified by grouping the technologies based on their use cases, namely information portal, digital health engagement, digital inclusion support, telehealth, telemedicine, care home technologies, booking system, online triage, and digital mental health services.

**Table 1:** Digital technologies used in NHS^a^ CCG^b^ and associated themes.

Digital options theory construct and theme		Example of technology
**Identifying patients’ requirements that involve recognizing new technical features for the digital health service**		
	Information portal	Webinar, health care promotion campaigns (eg, through social media), service direction (eg, via a web portal collating quick access to available digital health services), advice and guidance (eg, for GP^c^ to use to contact health specialists for advice), patient access to individual health care records, and fast information sharing portal (eg, eHealthscope, the development of which was led by Dr Michael O’Neil at the Saxon Cross Surgery, in partnership with local practices and the data management team of the 6 Nottingham CCGs)
	Digital health engagement	Digital champions (eg, to improve digital literacy), podcasts, and online community engagement about health care (eg, online meetings and surveys)
	Digital inclusion support	Technology for the digitally excluded (eg, providing devices for care homes) and alternative communication methods (eg, text messages)
**Developing new work patterns that improve internal coordination and working process**		
	Telehealth	Virtual assistants (eg, Alexa; Amazon Inc [22]), virtual wards, remote monitoring, and self-management software
	Telemedicine	AI^d^ diagnostics, teledermatology, digital prescribing (eg, OptimiseRx [23]), and applications using VR^e^ headsets
	Care home technologies	Digital technologies designed specifically for care homes and digital training tools for using these technologies for care home residents or staff
**Realizing improvements in efficiency and public accessibility**		
	Booking system	Indirect online booking (eg, the patient will need to submit a request form first and wait for an appointment) Direct online booking (eg, the patient can directly check available appointments and book one online)
	Online triage	Live online consultation and eConsult (eg, offline consultation system using a patient-filled form)
	Digital mental health services	IAPT^f^ (it is undergoing a national rebranding and will be called the NHS Talking Therapies for Anxiety and Depression), region-specific mental health service (eg, Mind-BLMK^g^ [24] for people in Bedfordshire, Luton, and Milton Keynes, With Me In Mind [25] for people in Doncaster, Rotherham, and North Lincolnshire), and independent mental health care (eg, Kooth; Kooth PLC)

^a^NHS: National Health Service.

^b^CCG: clinical commissioning group.

^c^GP: general practitioner.

^d^AI: artificial intelligence.

^e^VR: virtual reality.

^f^IAPT: Improving Access to Psychological Therapies.

^g^BLMK: Bedfordshire, Luton, and Milton Keynes.

#### Cluster Analysis

Hierarchical clustering [26] with Euclidean distance was used to extract common patterns from the code; by doing so the CCGs implementing similar types of technologies were grouped together. We used the identified 9 digital technology themes from the previous step to cluster the CCGs, in which the number of technology types mentioned for each theme was assigned to each CCG. For example, the Sheffield CCG reported they only had 1 technology (remote monitoring) under the telehealth theme, therefore the value of telehealth for Sheffield CCG was 1. In total, for each CCG, there were 9 values describing how engaged the CCG was with each technology theme. These values ere further standardized using the *z* score to achieve balanced similarity weights across all 9 themes. The silhouette and elbow method were used to select the optimal numbers for clusters [27], resulting in a structure with 3 clusters.

### Ethical Considerations

This study is based on the secondary analysis of NHS CCG annual reports, which are public information. The ethics approval for the secondary analysis of all data presented in this study was obtained from the University of Sheffield Research Ethics Committee (045790).

## Results

### Digital Technologies in NHS CCGs

#### Overview

In total, 18,667 pages from 135 annual reports were preprocessed and 2163 pages containing selected keywords were highlighted in the 135 reports, representing 106 CCGs. Further, 31 types of technologies were then extracted, covering a wide range of tools and purposes. These technologies were then grouped into 9 themes. The full codebook can be found in Multimedia Appendix 1.

The purposes of the digital technologies are typically associated with the patients’ or health care practitioners’ needs across various use cases and regions. Generalizing across all the observed items, these technologies were first grouped under particle themes, specific to either a location (eg, care home), disease (eg, mental health), or a type of service (eg, booking appointment). We then further assigned the identified 9 themes to 1 of the 3 digital options theory constructs. Table 1 describes the digital technologies observed from the NHS CCG annual reports and their associated themes.

#### Information Portals

The information portal theme consisted of 6 technologies that all involved access to or communication specific health care information via an online format. In total, 72% (76/106) of CCGs contained a mention of this theme via either 1 or more of the 6 technologies. Health care promotion for raising the public’s awareness of available services (35/106, 33%) was the most popular method, followed by online service catalogues (30/106, 28.3%) and webinars designed to briefly or formally announce digital tools (30/106, 28.3%). Providing patients with their own health care records accounted for 6% (7/106). These 4 above all involve having health care information readily available online for patients to locate and use. Other tools were typically more oriented toward the sharing of health care information between health care professionals or to the patients. For example, advice and guidance (16/106, 14.8%) was a tool that allowed general practitioners (GPs) to contact specialists quickly to obtain information for a patient so they did not have to refer the patient to the specialist. In doing so they saved time and could provide appropriate treatment for the patient on the same day. The health practitioners could also share electronic patient records quickly with relevant members through an online system (47/106, 43.5%) to facilitate discussion.

#### Digital Health Engagement

Digital health engagement was about engaging with the public through digital means (67/106, 63.2%). Differing from the information portal theme aiming to support patients or health practitioners dealing with immediate health needs, this theme focuses on obtaining feedback on service delivery and raising awareness of a disease or healthy lifestyle in the community in a less formal manner. The most popular technologies within this category involved patients in the operations of the health care service within their area through virtual meetings or surveys (63/106, 59.4%). These activities kept patients informed and created and allowed them to give their input on the operations of the health care service. Around 12% (13/106) of the reports discussed the development of a podcast that patients could access anytime that kept them up to date with any health care developments or gave them advice for better self-care management. Around 17.9% (19/106) of reports mentioned the use of digital champions, who are individuals who help staff and patients struggling with the integration of digital technologies and help them develop their digital skills and confidence.

#### Digital Inclusion Support

The implementation of many digital technologies was vital to keeping the NHS operating during the COVID-19 pandemic, and there was a conscious effort to keep providing adequate health care to those without a means of accessing these services. In total, 42.5% (45/106) of CCGs included recognition of the problem of digital exclusion and how they would tackle it via an alternative digital approach. The Newcastle Gateshead CCG report reflected on the downside of the increase in digital technology, suggesting that it could impact the NHS’ free at point of care policy (eg, using a phone or the internet is not free). Methods such as providing targeted alternative communication methods (eg, text message or physical newsletters; 14/106, 13.2%) or providing essential technology (typically providing hardware to care homes; 38/106, 35.5%) were mentioned.

#### Telehealth

Telehealth is a theme that aims to incorporate digital technologies to monitor patients’ health information through real time data and provide long-distance health care. At least one form of telehealth was mentioned by 82% (87/106) of the CCGs and was seen by many of them as vital to providing safe health care during the COVID-19 pandemic. Remote monitoring (69/106, 65.1%) typically involved the use of software or hardware technologies such as pulse oximetry and digital blood pressure monitors. The information from these devices would automatically be sent to the patients’ health care record, which allowed the GP to continuously check the patient’s vitals. This allowed the GP to take quick action as needed as they would be notified if any major problems arose. Further, 26.4% (28/106) of the reports mentioned that telehealth tools also helped patients with self-management, particularly for patients with chronic conditions. In addition, this theme involved some innovation and creative use of technology by some CCGs. For example, 6.5% (7/106) of the reports discussed providing Alexa (Amazon Inc) devices to patients who were not able to use traditional computer devices (due to conditions such as vision impairments). The speech recognition software would allow the patient to keep in contact with their GPs, where they could also be monitored remotely. We note that some reports mentioned the use of telehealth tools in general terms rather than naming the specific technology implemented (such as the name or provider of the tool).

#### Telemedicine

Telemedicine was one of the least mentioned themes among the CCG groups, with it only being mentioned by 33% (35/106) of CCGs. Telemedicine differs from telehealth in that it seeks to provide treatment or make a diagnosis for patients using digital technologies. The more innovative approach to digital technologies would lie within this category. This includes health care practices using virtual reality headsets (2/106, 1.9%) to treat patients for their mental health by having them experience scenarios in a digital space. AI diagnostics, mentioned by 9% (10/106) of reports, makes predictions about a patient’s health (eg, heart-related issues or developing cancer), allowing the health care provider to advise a patient so they may mitigate any future health care concerns. We also found that 10.3% (11/106) of the reports mentioned technologies for online prescribing, often for a repeat prescription. Teledermatology, mentioned by 7.5% of the reports (8/106), refers to the use of static digital images to triage, diagnose, monitor, or assess skin conditions without the patient physically meeting the dermatologist. This technology required the users to submit a clear photo of their skin condition before the appointment (either in the form of online meetings or chat). This dependency on trust in technology (so that the users submit personal information through the tool and believe in the results) as well as effort in the form of hardware (such as a virtual reality headset, laptop, or mobile camera for taking pictures and making video calls) from the users, is consistent for all tools in this theme.

#### Care Home Technologies

We refer to care home technologies as digital technologies designed specifically for the care home or training tools for care home residents and staff to use digital technologies. The technologies under this theme contained accessibility considerations particularly designed to support people in the later stage of life and living in care homes; therefore we separated it as a single theme. Care home technologies were only mentioned by about 37.7% (40/106) of the reports. By 2021, it was predicted that more than 400,000 people would live in care homes in the United Kingdom and would likely need close monitoring and more delicate health care due to age or health conditions than older people staying at home. In total, 35.8% (38/106) of the CCGs mentioned the involvement of training staff within these settings so they could adequately use the new digital technologies to care for their residents.

#### Booking System

In total, 24.5% (26/106) of CCGs had started to adopt various forms of online booking systems, especially within GP practices. This is the alternative to the traditional method of a patient telephoning a GP asking for an appointment through these means. The reasons cited for the uptake of this theme by some CCGs can be attributed to three factors: (1) the integration of NHS 111 (a free-to-call single nonemergency number medical helpline) services with GP and emergency departments (EDs), (2) the desire to ease the burden on telephone lines, and (3) the development of the NHS app. Further, 2 types of technologies, namely indirect booking and direct booking, were found within this theme. Indirect online booking was mentioned by 13.2% (14/106) of the CCGs, and this was the process of NHS 111 having the ability to book patients directly into GP or ED appointments. Multiple reports mentioned that the sharing of data between services (such as GPs, NHS 111, and ED) allowed NHS 111 to filter patients through their lines and book patients into appointments that they urgently needed. For example, the Manchester CCG used the Adastra Digital solution to send information between EDs and NHS 111. This improved patient flow and eased the burden on GPs and EDs. Direct online booking is when the patient themselves can book, manage, and also cancel their own appointment online. This was mentioned by 14% (15/106) of the CCGs and many of them cited the integration of the NHS app as a convenient means of allowing patients access to book appointments themselves.

#### Online Triage

Virtually every CCG (104/106, 98.1%) mentioned some form of online triage that was integrated into their effort to adopt digital technologies. This mostly came from the reports covering the use of virtual consultations by their health care professionals. Many reports discussed how the COVID-19 pandemic forced them to accelerate their plans to integrate digital solutions into their health care plans, and virtual consultations became a must for many health care settings so they could continue operating safely (both for the practitioner and for the patients). Typically, this involved practitioners contacting patients via telephone or some form of video consultation services. The video consultation would usually be operated by private health care platforms such as Doctorlink (HealthHero), askmyGP (Evergreen Health Solutions Ltd), or Attend Anywhere (Induction Healthcare Group PLC), in which case the private companies created a process to help practitioners carry out this health care service (either through a telephone call, a smartphone app, or a web app). eConsult is a form of online triage mentioned by 19.6% (21/106) of CCGs and differs from the virtual consultation format as it involves patients filling out an online form and sending it to their health care practice, where it is reviewed and next steps for treatment are provided by a health care professional without directly speaking to the patient. This service was cited as being useful for prescribing repeat prescriptions and removing pressure on phone lines.

#### Digital Mental Health Services

Digital mental health services were popular among CCGs, being mentioned by 70.3% (74/106) of them. We decided to put these technologies into a separate theme due to the increased mental health needs during the pandemic and the innovations used to carry out traditional treatments (such as psychotherapy or counseling). Some mental health treatments involved using digital tools, such as video calls, to offer counseling services before the pandemic. These mental health services often incorporated other digital technologies such as information portals (eg, Instagram accounts) to share advice and tips for self-management. Around 38.7% (41/106) of CCGs developed their own region-specific digital mental health care services that they were able to refer patients to. For example, Mind-BLMK is available for people in Bedfordshire and Luton as well as Milton Keynes, and With Me in Mind is available for people in Doncaster, Rotherham, and North Lincolnshire. However, around 33.9% (36/106) of CCGs discussed how they had integrated private mental health services that they had partnered with to refer their patients to. Kooth was one of the more popular services as it focused on treatment for children and younger people, although other services such as Qwell [28] specialized in treatment for adults. Both sites are similar in layout, and they tailor their services to the targeted user group. For example, Kooth offers the opportunity for young people to engage in mini activities to manage their mental health, while Qwell offers more traditional long-form articles that explore different mental health issues and solutions. Both sites also offer online forums to talk to other patients of a similar age using the services, potentially sharing experiences and offering peer support to build a community between each other. Both sites offer access to therapists who specialize in the targeted age groups, as well.

### Clusters

Clustering was used to identify groups of CCGs implementing similar types of technologies. We first calculated Hopkins statistics for the data. The Hopkins statistic was 0.9914 (>0.5), indicating the data were highly clustered. A structure with 3 clusters was the optimal cluster structure (average silhouette width of 0.186, SD 0.163), determined using the silhouette and elbow method [27].

**Figure 1:**
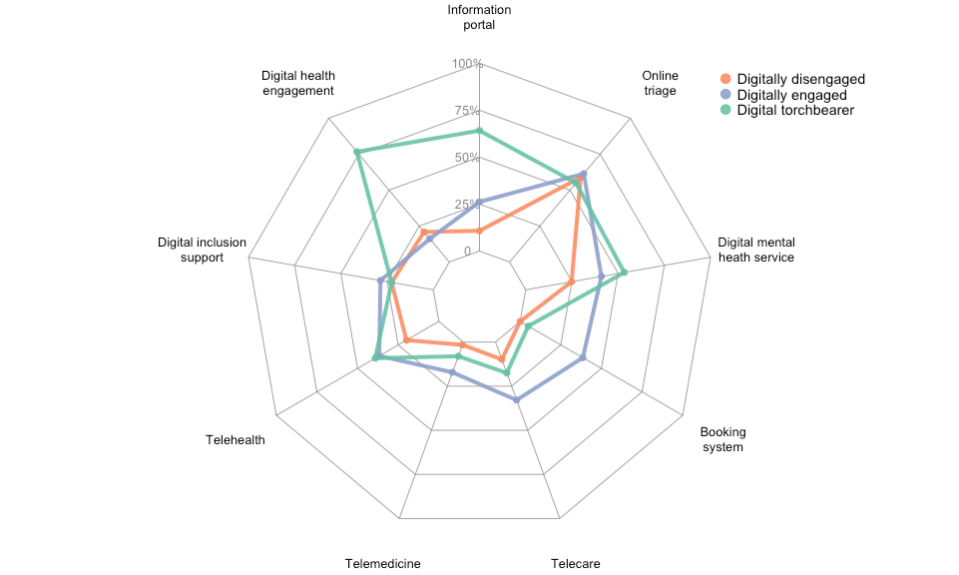
Three clusters and their scores for each digital technology theme.

Resulting from hierarchical clustering, the first cluster group contained 51 CCGs, the second cluster contained 35, and the third contained 20. The CCGs differed between clusters in their digital themes. Figure 1 helps establish the differences between each group by showing the weighted proportions of technologies in each theme aggregated across CCGs in each group, which we refer to as “scores” in the following text and Figure 1. There were 9 scores assigned to each CCG, each corresponding to a digital theme. Group 1 (orange) will be named “disengaged” (of digital technologies) due to low scores in all of the digital themes within the CCG reports within this cluster (8 out of 9 themes had scores less than 25%). Cluster 2 (blue) will be named “engaged” (with digital technologies) due to the CCG reports within this cluster having a general interest in many of the digital themes (6 out of 9 themes had scores between 25% and 50%). This is especially true for telemedicine ,where it excels compared to the other two clusters; however, there is a low score for the digital engagement and telemedicine themes. Group 3 (green) did not have this problem as the CCG reports within this cluster had a high score for technologies in the digital engagement category, as well as a very high score for those in the information portal category. Therefore, this group will be named “torchbearer” (of digital technologies).

**Figure 2:**
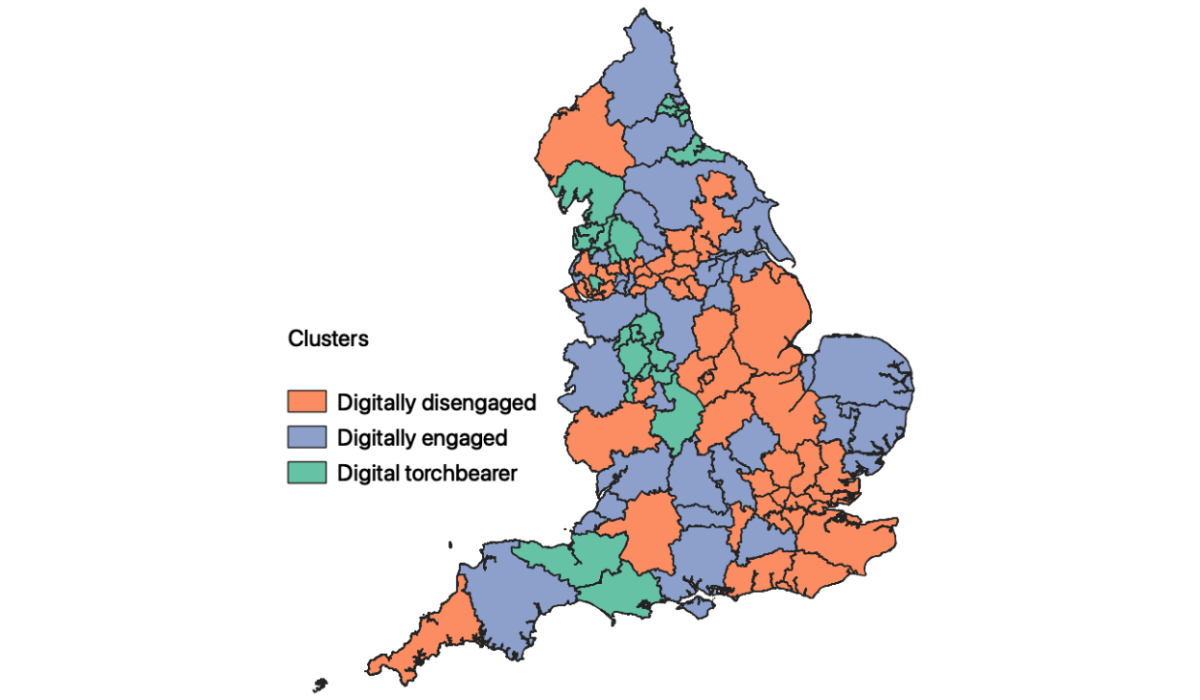
The distribution of cluster groups in England.

Table 2 presents the number of CCGs in each cluster group represented by the region they belong to according to the UK Office for National Statistics. For example, in the southeast region, there were 5 CCGs that belonged to the digitally disengaged cluster and 6 CCGs that belonged to the digitally engaged cluster. Figure 2 displays the geographical distribution of cluster groups in England. The graph does not present a particular spatial pattern, but there are some notable observations. For one, all of the London CCGs fell into the digitally disengaged group. The northwest and Midlands regions have a proportionally very low presence in the digitally engaged cluster group. This may be due to them having a very high presence in the digital torchbearer group.

**Table 2:** Regional distribution of cluster groups (n=106) (number of boroughs in each region).

Cluster groups	London, n	Southeast, n	Southwest, n	East of England, n	Midlands, n	Northeast and Yorkshire, n	Northwest, n
Disengaged	5	5	2	9	8	9	13
Engaged	0	6	3	5	3	11	7
Torchbearer	0	0	2	0	7	4	7

## Discussion

### Principal Results

The pandemic has disrupted health care support services in the United Kingdom, resulting in the rapid adoption of digital technologies [29]. Nevertheless, the digital technology themes guiding the mass adoption of technology and the potential regional disparities remain to be explored. Based on 135 NHS CCG annual reports, this paper identified digital technology themes and associated technologies adopted by the NHS nationally in 2020 and 2021 and examined their regional differences from a digital divide perspective [30].

Informed by digital options theory [16,17], we identified 9 digital technology themes, which were categorized into 3 main groups, namely, the identification of patients’ requirements, the development of new work patterns that improve internal coordination and working process, and realizing improvements in efficiency and public accessibility. First, the identification of patients’ requirements includes the use of information portals (eg, retrieving and sharing health-related information), digital means (eg, using podcasts for promoting health-related information), and digital support (eg, providing hardware or alternative access for the digitally excluded) to achieve effective communication channels between health care providers and patients. Second, the development of new work patterns that improve internal coordination and working process includes digitizing existing health care services, such as remote monitoring of patient health (ie, Alexa), e-prescriptions (eg, AI diagnostics and online prescriptions), and digital training (eg, staff training on using digital tools in care homes). Finally, the realization of improvements in efficiency and public accessibility results in the use of digital methods to widen public access to health care, including creating online booking systems shared by health care providers (eg, NHS 111 and NHS apps), online triage (eg, online consultation), and online mental health services (eg, Kooth). Our data suggest that there are prominent digital themes within each group. Information portals are mostly adopted by CCGs to achieve effective health care communication among patients and health care providers. Telehealth is primarily adopted by CCGs for digitizing health care services, particularly to monitor patient health remotely. Online triage has been widely implemented by CCGs to provide patients with access to health care during the pandemic. Therefore, these top 3 themes, which cover people’s fundamental health needs, could serve as a starting point for future CCGs and other health care providers when adopting and implementing digital solutions.

In addition, our findings contribute to the new research theme of “digital health citizenship” by highlighting that digital tools and technologies extend beyond operation efficiency to wider patient engagement, and therefore reshape social relations and interactions among patients as health service users [2]. Regional differences in such social relations and interactions could be linked to the equality and inclusion issues in health service provision. Based on the 9 identified digital themes, 3 main clusters were identified: the digitally engaged, the digitally disengaged, and the digital torchbearer. It is concerning that almost half of the CCGs fell into the digitally disengaged group, showing a low uptake of the aforementioned digital themes. Interestingly, most digitally disengaged CCGs belonged to London areas. This seems to be aligned with the data released by the Office for National Statistics [31] in 2019, suggesting that London has the lowest percentage of internet nonusers in the United Kingdom by population. However, Watson et al [32] also pointed out that a lack of digital devices and private spaces for accessing online health care could also act as barriers to digital health care themes in London. The use of such digital devices and online health care services opens up a new set of digital rights, opportunities, and responsibilities for patients [2], and this needs to be balanced across regions to ensure the equality and inclusion of health care. Our data suggest that CCGs are still not sufficiently efficient when it comes to adopting digital themes.

Furthermore, our findings highlight the impact of COVID-19 on the development of a CCG’s digital technology themes. Many of the reports cited COVID-19 as accelerating the need to digitize health care. For example, the use of telehealth to increase remote monitoring of patients or encourage them to manage their own health increased during the pandemic due to patients not being able to reach their own health care practices as freely. Most of the reports were generally very positive about increasing the inclusion of digital technology. Reasons for the positives statements included making it easier to access health care, providing early treatment, providing more data to improve services, and making the health care practice more agile and efficient. However, some reports cited reasons for concern about increasing digital health care when discussing the possibility of digital exclusion for some patients. Further, one report from the Newcastle Gateshead CCG raised concerns about how the integration of digital technology may impact the NHS policy of being free at point of use. This issue is consistent with concerns expressed by other researchers. For example, Clare [33] and Eruchalu et al [34] have noted the potential limitations of telehealth and telemedicine due to broadband connectivity issues resulting from socioeconomic disparities among regions, particularly among the underprivileged, the medically underserved, and in communities of color. With services becoming increasingly online (eg, many mental health services were all online) and not everyone having access to technology, there is a justifiable concern. To realize equitable benefits from health-related technologies across all populations, it is imperative to thoroughly examine and address complex issues, such as social, cultural, and economic factors that hinder accessibility and adoption among different communities. Otherwise, as pointed out by Ramsetty and Adams [35], despite technological advancements, disparities in health care access and outcomes will inevitably persist, particularly among the most vulnerable during times of crisis. Future research could further investigate this area and how these issues could be addressed.

It is essential to highlight that the absence of digital technology mentioned in a CCG report does not necessarily indicate a failure in its adoption. For example, eConsult is an online triage form that patients can fill out and send to their health care practitioner where it is reviewed and the next steps for treatment are accessed by a health care professional for the patients to take. The Kent and Medway CCG did not make any reference to this digital technology within their annual report [36], however, when investigating individual GP practices that operate under them, the technology is being used. This suggests that the lack of mention of a particular technology by a CCG group does not mean it is not being used at all but indicates the action of using such technology is not applied at the CCG level.

### Conclusion

This research mapped out the current digital technology themes adopted by CCGs in the United Kingdom when providing health care services during the pandemic. These identified themes can be used by future health care providers to adopt digital solutions to address different health care issues, such as improving communication, digitizing their existing services, and increasing public access to health care. Furthermore, the research highlights the existence of a digital divide within CCGs in terms of adopting digital technology themes, particularly when it comes to regional disparities. The possible solutions could include providing support to the “digitally disengaged” CCGs in using various identified digital technology themes and becoming more digitally “active” or “engaged.” Caution needs to be taken when offering support as well. For example, we need to fully explore and understand the reasons behind their slow uptake of digital technologies in order to offer tailored solutions. To build upon our findings and promote equal access to digital health benefits for all communities, future research could examine the factors that contribute to regional disparities in digital technology adoption and access within and across CCGs. A comprehensive understanding of the underlying causes of these disparities could help policy makers and health care professionals focus their efforts more effectively toward bridging the digital divide and improving access to health resources and support for the public.

Although our study offers great insights into the digital technology themes adopted by CCGs, there are a few limitations that need to be addressed. Our findings are mainly based on CCG reports. The length, focus, and description details may differ slightly between these reports. In addition, there may be underreporting when it comes to digital technology themes by individual CCGs, resulting in misrepresentation when analyzing reports. Finally, the research focus was on the digital technology themes and the adoption of associated technologies rather than the impact these technologies had on patient satisfaction or patient outcomes. Future research could look at the impact of these identified digital technology themes or individual technologies and their effectiveness through in-depth interviews or questionnaire surveys with patients and health care professionals.

## Appendix

Multimedia Appendix 1 Codebook.

## Abbreviations

CCG: clinical commissioning group
ED: emergency department
GP: general practitioner
ICB: integrated care board
NHS: National Health Service

## Data Availability

All data analyzed during this study are included in this published article and its supplementary information files.
